# Extended-Spectrum β-Lactamase- and Carbapenemase-Producing Enterobacteriaceae Isolated from Egyptian Patients with Suspected Blood Stream Infection

**DOI:** 10.1371/journal.pone.0128120

**Published:** 2015-05-22

**Authors:** H. M. Abdallah, B. B. Wintermans, E. A. Reuland, A Koek, N. al Naiemi, A. M. Ammar, A. A. Mohamed, C. M. J. E. Vandenbroucke-Grauls

**Affiliations:** 1 Medical Microbiology and Infection Control, VU University Medical Center, Amsterdam, the Netherlands; 2 Department of Bacteriology, Mycology and Immunology, Faculty of Veterinary Medicine, Zagazig University, Zagazig, Egypt; 3 Laboratory for Medical Microbiology and Public Health, Hengelo, the Netherlands; 4 Microbiology and Infection Control, Ziekenhuisgroep Twente, Almelo, Amsterdam, the Netherlands; Second University of Naples, ITALY

## Abstract

**Objectives:**

The aim of the study was to investigate the prevalence of extended-spectrum β-lactamase and carbapenemase production among Enterobacteriaceae isolated from Egyptian patients with suspected blood stream infection.

**Methods:**

Ninety-four Enterobacteriaceae blood culture isolates from Egyptian patients with suspected blood stream infection were collected, one isolate per patient. Identification of bacterial isolates was performed with MALDI-TOF (MS-based Vitek MS system, bioMerieux). Screening for ESBLs and carbapenemases production was done with the Vitek 2 system (bioMérieux). ESBL production was confirmed using the combined disk diffusion method for cefotaxime, ceftazidime, and cefepime, all with and without clavulanic acid (Rosco). Real-time PCR and sequencing were used to characterize the resistance genes. The phylogenetic groups of *E*. *coli* were identified by a PCR-based method.

**Results:**

Of the 94 Enterobacteriaceae isolates 46 (48.93%) showed an ESBL phenotype. One *Enterobacter* spp isolate was ESBL-producer and meropenem-resistant. The genetic analysis showed that CTX-M was present in 89.13% (41/46) of the ESBL-producing Enterobacteriaceae, whereas TEM and SHV were detected in 56.52% (26/46) and 21.74% (10/46) respectively (47.83%) of the ESBL-producing isolates were multidrug resistant (MDR). Eleven out of 30 ESBL-producing *E-coli* isolates were assigned to phylogroup B2, followed by groups B1 (8 isolates), A (6 isolates) and D (5 isolates).

**Conclusions:**

The high ESBL-E rates (48.93%) found in this study together with the identification of one carbapenem-resistant *Enterobacter* spp isolate is worrisome. Our results indicate that systems for monitoring and detection of ESBL-producing bacteria in Egyptian hospitals have to be established. Also strict hospital infection control policies with the restriction of the consumption of extended-spectrum cephalosporins are necessary.

## Introduction

The β-lactam antibiotics are the most commonly used therapeutic class of antimicrobials for treatment of bacterial infection because of their broad antibacterial spectrum and excellent safety profile [1]. Bacterial resistance to antibiotics is increasing worldwide in healthcare settings and in the community [2]. Resistance to β-lactam antibiotics can be caused by either the production of β-lactamase enzymes, the presence of β-lactam-insensitive cell wall transpeptidases, or the active expulsion of β-lactam molecules from Gram-negative cells by efflux pumps [3]. In Enterobacteriaceae, β-lactamase production remains the most important mediator of β-lactam resistance [4]. Extended-spectrum β-lactamases (ESBLs) are a rapidly evolving group of β-lactamases which hydrolyze the extended-spectrum cephalosporins, the penicillins, as well as aztreonam, but not carbapenems [5,6].The spread of extended-spectrum β-lactamase-producing Enterobacteriaceae (ESBL-E) is increasing worldwide [7]. They cause community- and hospital-associated infections in both humans and animals [8]. Carbapenems have served as the first-choice drugs for the treatment of ESBL-E especially with the increasing reports of ESBL-producing clinical isolates expressing multidrug resistance (MDR) [9]. The emergence of carbapenemases—producing Enterobacteriaceae (CPE), is causing an unprecedented public health threat leaving few treatment options. CPE not only infect hospitalized patients, but have also spread in long-term care facilities [10]. In Egypt, information about the prevalence of ESBL-E is limited. According to the Pan European Antimicrobial Resistance Local Surveillance (PEARLS) study (2001–2002), performed in 13 European, three Middle Eastern countries and South Africa, Egypt was one of the countries with the highest rate of ESBL among Enterobacteriaceae (38.5%) [11].A more recent surveillance program for nosocomial bloodstream infections revealed that ESBL production was found to be 80.6% among *K*. *pneumoniae* and 40.9% among *E-coli* [12]. Moreover, the majority of CTX-M-15-producing *E-coli* was related to the international clonal complex ST131 [13]. CPE was also recently reported in Egypt [14]. The aim of this study was to investigate the prevalence of extended-spectrum β-lactamase and carbapenemase production among Enterobacteriaceae isolated from Egyptian patients with suspected blood stream infection.

## Materials and Methods

### Bacterial isolates

This is study was conducted in the period between January 2013 and May 2013, at El-Ahrar General Hospital, Zagazig, Egypt, a 608-bed hospital affiliated to the Egyptian health ministry. 10 ml of blood was collected aseptically by peripheral venipuncture from every patient with suspected blood stream infection. Blood culture was done by conventional method described elsewhere [15]. A total of 94 enterobacterial isolates, one isolate per patient were identified by the automated Vitek MS system (BioMérieux, Marcy l’Étoile, France).

### Phenotypic screening and confirmation of ESBL-E and CPE

The antimicrobial susceptibility testing was performed with the VITEK2 system with AST N198 (BioMérieux, Marcy l’Étoile, France). The interpretation of antimicrobial susceptibility test results followed recommendations of European Committee on Antimicrobial Susceptibility Testing (EUCAST) [16]. Phenotypic ESBL production was confirmed with the combination disc diffusion test with clavulanic acid (Rosco, Taastrup, Denmark) [17]. The inhibition zone around the cephalosporin (cefotaxime, ceftazidime and cefepime) tablet combined with clavulanic acid is compared to the zone around the tablet with the cephalosporin alone. The test is positive if the inhibition zone is ≥ 5 mm larger with clavulanic acid than without [17].

Carbapenemases production was confirmed by carbapenemases double disk synergy test [18]. Enhancement of the inhibition zone in the area between the carbapenems (meropenem and /or imipenem) and the inhibitor-containing disk (boronic acid and/or dipicolinic acid) was considered to be a positive result [17,19].

### Real-time PCR for characterization of β-lactamase-encoding genes

All phenotypic ESBL producers were screened by real-time PCR to identify their ESBL-carrying genes with specific primers for TEM [20], SHV [21], and CTX-M [22]. Phenotypic carbapenemases were analyzed for the presence of genes encoding KPC, NDM, OXA-48, IMP, and VIM by multiplex PCRs using primers described before [23]. DNA was extracted by a boiling lysis method as described [24]. All real-time PCR amplifications and melting curve analysis were carried out on the LightCycler 480 II system with software version 3.5 (Roche) in a total volume of 20 μl. Amplification conditions were described elsewhere [25,26].

### DNA sequencing analysis

The amplicons of ESBLs producers were sequenced with the Sanger ABI 3730 XL automated DNA sequencer (BaseClear, Leiden, The Netherlands). The nucleotide sequences were analyzed using the Codon Code Aligner software (Version 5.0.2) and were compared to sequences available at the National Center for Biotechnology Information website (www.ncbi.nlm.nih.gov).

### Determination of E-coli Phylogroups

The assignation of the *E*. *coli* isolates to one of the four main phylogenetic groups (A, B1, B2, or D) was performed by a PCR-based method targeted to the *chuA*, *yjaA* genes and the *TspE4*.*C2* DNA fragment, as developed by Clermont et al.[27].

### Ethics Statement

Formal permission was obtained from the managers of Al Ahrar hospitals. Informed written consent was obtained from all participants in this study after explanation of the procedure and the purpose of the study. The study was approved by the review boards of the Research Ethics Committee, Faculty of Medicine, Zagazig University

## Results

Of the 94 tested clinical strains of Enterobacteriaceae isolated from blood of Egyptian patients with suspected blood stream infection, 46 (48.93%) were ESBL positive.

The frequency of ESBL production per species among the tested isolates was the following: 54.5% (30/55) *E*. *coli*, 66.66% (10/15) *Klebsiella pneumoniae*, 35.71% (5/14) *Enterobacter* spp, and one out of 2 isolates of *Morganella morganii*. One *Enterobacter aerogenes* isolate was ESBL-producer and resistant to both imipenem and meropenem.

The genetic analysis showed that CTX-M was present in 89.13% (41/46) of the ESBL-producing Enterobacteriaceae, whereas TEM and SHV were detected in 56.52% (26/46) and 21.74% (10/46) respectively. A summary of the different types of β-lactamase-encoding genes among different Enterobacteriaceae is provided in Table 1.

**Table 1:** Prevalence of the different types of β-lactamases among different Enterobacteriaceae species.

	*E*.*coli*	*Klebsiella pneumoniae*	*Enterobacter spp*	*Morganella morganii*	Other *species*	Total
CTX-M-15 alone	15	0	0	0	0	15
CTX-M-15 + TEM1	12	0	1	0	0	13
CTX-M-14 + TEM1	2	0	0	0	0	2
CTX-M-15 + CTX-M-14 + TEM1	0	0	1	0	0	1
CTX-M-14 + SHV1	0	2	0	0		2
CTX-M-15 + SHV11	0	1	0	0	0	1
CTX-M-15 + SHV12	0	1	0	0	0	1
TEM1 alone	1	0	3	0	0	4
CTX-M-15 + TEM1 + SHV1	0	2	0	0	0	2
CTX-M-15 + TEM1 + SHV11	0	3	0	0	0	3
CTX-M-15 + TEM1 + SHV12	0	1	0	0	0	1

Sequence analysis revealed that CTX-M comprised mainly CTX-M-15 (37/41, 90.24%) and CTX-M-14 (5/41, 12.20%). One *Enterobacter* spp isolate harbored both alleles. All TEM positive isolates were found to harbor the prototype TEM-1 enzyme, while SHV belonged to SHV-1 (n = 4), SHV-11 (n = 4), and SHV-12 (n = 2).

One *Morganella morganii* isolate expressed ESBL phenotype but no TEM, SHV or CTX-M was detected by PCR. The carbapenem resistant *Enterobacter aerogenes* isolate was found to be positive for NDM.

Of the 46 ESBL-producing isolates, 37 (80.43%) showed combined resistance to quinolones (ciprofloxacin and /or norfloxacin), 36 (78.26%) to trimethoprim/sulfamethoxazole, 30 (56.22%) to aminoglycosides (gentamicin and/or tobramycin) and 9 (19.57%) to nitrofurantoin. Twenty-two (47.83%) of the ESBL-producing isolates were multidrug resistant (MDR) (i.e. not susceptible to at least one agent in three or more classes of antimicrobials (aminoglycosides, quinolones and cotrimoxazole) [28].

Phylogenic analysis revealed that 11 out of 30 ESBL producing *E-coli*isolates belonged to phylogroup B2, 8 to group B1, 6 to group A, and 5 to group D. The distribution of the different ESBL types over the phylogroups is shown in Table 2.

**Table 2:** Phylogenetic groups of ESBL producing *E-coli* isolates.

Type of ESBL	Number of isolates belonging to phylogenetic groups			
	A	B1	B2	D
CTX-M alone (n = 15)	2	5	7	1
CTX-M +TEM (n = 14)	4	3	4	3
TEM alone(n = 1)	0	0	0	1
Total(n = 30)	6	8	11	5

## Discussion

This study was carried out to determine the prevalence of extended-spectrum β-lactamase and carbapenemase production among Enterobacteriaceae isolated from Egyptian hospitalized patients with suspected blood stream infection. Our findings showed that 48.93% of enterobacterial strains isolated from blood specimens were ESBL producers. This is a high prevalence compared to many European countries (http://www.ecdc.europa.eu/en/healthtopics/antimicrobial_resistance/database/Pages/database.aspx). Possibly, this high prevalence is related to the less controlled use of antibiotics in Egypt, where many drugs are still available over the counter. One carbapenem-resistant strain was detected.

Few studies have investigated the prevalence of ESBL-E in Egyptian hospitals. Bouchillon et al conducted the PEARLS study in 2001–2002, and found that 38.5% of Enterobacteriaceae isolates did produce an ESBL [11]. A lower ESBL prevalence rate (16%) was found among 120 isolates collected between May 2007 and August 2008 at the Theodor Bilharz Research Institute, Cairo, Egypt [13]. In 2009, Ahmed et al detected 64.7% ESBL-producing Enterobacteriaceae among strains isolated from patients in the intensive care unit of a university hospital [29]. Considering that we included bloodstream infections from all departments in El-Ahrar General Hospital, Zagazig, Egypt, the rate that we observed is in line with the findings of Ahmed et al.

The results obtained in this study showed that *bla*_CTX-M_ type was the most prevalent β-lactamase-encoding gene. It was detected in almost 90% of the ESBL-producing Enterobacteriaceae, whereas *bla*_TEM_ and *bla*_SHV_ were present in about half and one fifth of isolates, respectively. These findings agree with other contemporary studies from around the world that show that ESBL genes of the CTX-M are dominant [30,31]. In contrast to our findings, Ahmed et al.[29]reported that *bla*_TEM_ was the most frequent β-lactamase-encoding gene.

One NDM-producing *Enterobacter aerogenes* isolate was found. This goes along with the recent detection of NDM-producing enterobacterial strains in Egypt, as well as other countries in the Middle East and North Africa [14,32–34].

Our data showed that the CTX-M-15 allele was the dominant CTX-M type ESBL with a frequency of over 90%followed by the CTX-M-14 allele with a frequency rate of over 12%. One isolate expressed the two alleles. These findings were concordant with the results of other studies carried out in Egypt and Europe [13,35,36].

Although TEM-1 and SHV-1 are not regarded as extended-spectrum β-lactamases, presence of these enzymes, combined with changes in the outer membrane proteins lead to reduced susceptibility to third-generation cephalosporins, that phenotypically suggest ESBL production [37]. Both SHV-11 and SHV-12 identified in this study are SHV-type ESBL [38]. SHV-12-producing *Klebsiella pneumoniae* has been recently reported in Egypt [39].

As expected, the ESBL-producing blood isolates showed a high frequency of co-resistance to quinolones, trimethoprim/sulfamethoxazole, aminoglycosides, and nitrofurantoin. Co-resistance was multiple in many cases, which means that nearly half of the isolates were multidrug resistant. This high level of multidrug resistance poses even more treatment problems than just the production of β-lactamases.

Phylogenetic analysis of CTX-M-producing *E*. *coli* isolates revealed that most isolates belonged to the extraintestinal pathogenic group B2.This finding is consistent with previous studies that showed the dominance of phylogroup B2 among CTX-M-producers, and further confirms that this phylogroup contains resistant and virulent clones [40,41]. On the other hand, a study carried out by Brangeret al.[42] demonstrated that CTX-M is present mainly in strains belonging to group D, while TEM was mainly found in *E*. *coli* with genetic background B2. In our strain collection, TEM-producing *E*. *coli* isolates were nearly equally divided over phylogroups A, B2 and D, while group B1 was less represented. The differences between Branger’s and our findings are probably related to the different geographical background of the patients from whom the strains were collected. They show that interpretation of genetic background in relation to plasmid-borne resistance genes is influenced by where and when a strain collection is assembled.

In conclusion, nearly half of Enterobacteriaceae isolated from blood in patients suspected of blood stream infection were ESBL producers. This high frequency of ESBL-E is worrisome. Our results indicate that systems for detection and monitoring of ESBL-producing bacteria in Egyptian hospitals have to be established. Also strict hospital infection control policies with restriction of the consumption of expanded-spectrum cephalosporins are necessary.
